# Y Chromosome Haplogroup Distribution in Indo-European Speaking Tribes of Gujarat, Western India

**DOI:** 10.1371/journal.pone.0090414

**Published:** 2014-03-10

**Authors:** Priyanka Khurana, Aastha Aggarwal, Siuli Mitra, Yazdi M. Italia, Kallur N. Saraswathy, Adimoolam Chandrasekar, Gautam K. Kshatriya

**Affiliations:** 1 Department of Anthropology, School of Applied Sciences, Dr. Harisingh Gour University, Sagar, Madhya Pradesh, India; 2 South Asia Network for Chronic Disease, Public Health Foundation of India, Delhi, India; 3 Department of Anthropology, University of Delhi, Delhi, India; 4 Valsad Raktdan Kendra, R.N.C. Free Eye Hospital Complex, Valsad, Gujarat, India; 5 Anthropological Survey of India, Southern Regional Center, Mysore, Karnataka, India; University of Oxford, United Kingdom

## Abstract

The present study was carried out in the Indo-European speaking tribal population groups of Southern Gujarat, India to investigate and reconstruct their paternal population structure and population histories. The role of language, ethnicity and geography in determining the observed pattern of Y haplogroup clustering in the study populations was also examined. A set of 48 bi-allelic markers on the non-recombining region of Y chromosome (NRY) were analysed in 284 males; representing nine Indo-European speaking tribal populations. The genetic structure of the populations revealed that none of these groups was overtly admixed or completely isolated. However, elevated haplogroup diversity and F_ST_ value point towards greater diversity and differentiation which suggests the possibility of early demographic expansion of the study groups. The phylogenetic analysis revealed 13 paternal lineages, of which six haplogroups: C5, H1a*, H2, J2, R1a1* and R2 accounted for a major portion of the Y chromosome diversity. The higher frequency of the six haplogroups and the pattern of clustering in the populations indicated overlapping of haplogroups with West and Central Asian populations. Other analyses undertaken on the population affiliations revealed that the Indo-European speaking populations along with the Dravidian speaking groups of southern India have an influence on the tribal groups of Gujarat. The vital role of geography in determining the distribution of Y lineages was also noticed. This implies that although language plays a vital role in determining the distribution of Y lineages, the present day linguistic affiliation of any population in India for reconstructing the demographic history of the country should be considered with caution.

## Introduction

India is the second most populous country in the world with a population of 1.21 billion [1]. About 4,635 different population groups are spread across the country [2]. Exorbitant as it may sound, existence of at least 50–60 thousand essentially endogamous groups has been reported in the country [3]–[4]. The present day Indian population is divided into tribal and non-tribal groups. Tribal populations constitute 8.2% of the total population [5] and are considered to be the indigenous populations of India [6]–[8]. The tribal groups of India belong to four broad linguistic families: Austro-Asiatic, Dravidian, Indo-European and Tibeto-Burman [9]. The Indo-European and Dravidian speaking populations are considered to be the major contributors to the development of Indian culture and society [10]. Both Austro-Asiatic and Dravidian speaking tribes belong to the primary pre-historic populations of India [7], [11]. Tibeto-Burman speakers also include many tribal populations but their geographical distribution is largely restricted to the North-Eastern region of India. India thus exhibits an enormous genetic, cultural and linguistic diversity which can partly be attributed to its position at the tri-junction: with Africa in the west, Eurasia in the north and the Orient in the east. The country's unique location gives rise to a great variety of environmental conditions and associated biodiversity [12] which in turn has attracted people from all over the globe.

Archaeological and paleontological evidence dating back to the middle and late Pleistocene era points towards early human occupation of the Indian subcontinent [13]–[16]. Similar evidence of antiquity of Indian populations has been established by genetic marker studies. The high levels of gene diversity and coefficients of gene differentiation obtained using autosomal markers for Indian populations are a testimony of both, the antiquity and the complex population structure, of this massive human conglomeration existing in the southern part of Asia [17]–[19]. Recent genetic studies based on mtDNA showing the high frequency of mitochondrial lineages with greater age, high diversity and wide distribution amply demonstrate the prehistoric existence of mankind on the Indian subcontinent [20]–[21]. Similar analyses of Y chromosome variation based on both bi-allelic and microsatellite markers have documented the existence of substantially deep rooted lineages among Indian populations buttressing the claim of early habitation of humans on the Indian subcontinent [22]–[25].

The unique and complex structure of the Indian population is attributed to the multiple waves of migration and the resultant gene-flow which occurred in the past [12], [26]. Some scholars have provided evidence to explain the origin and migration of the major linguistic families extant in India [25], [27]. It is worth mentioning that speakers of Austro-Asiatic language in India are exclusively tribal, which may be indicative of their being one of the oldest inhabitants of India [28], [29]. The arrival of the Indo-European speakers via the Northern corridor of India around 3,500–4,000 years ago is believed to be responsible for one of the major influxes of people in the Indian subcontinent [30], followed later by the infiltration of colonizers from different parts of the world. Thus, several studies have affirmed the influence of Eurasian and Asian populations on the Indian gene pool [23]–[24], [31]–[34].

Within this complex scenario, many interaction models such as gene-language, gene-geography and gene-ethnicity have been contested. Previous studies have shown inverse correlation between genetic affinities and geographical distance [11], [35]. Significant genetic differentiation between caste and tribal populations has been reported [31], [33], [36], as against a model which suggests that there is considerable sharing of Pleistocene heritage among them with a limited gene flow [34]. Similarly, congruence between language and genes has been proposed by various scholars [9], [37]–[38] along with a competing view supporting that genetic affinities may not necessarily be dependent on linguistic similarities [39]–[41]. Although each study has contributed significantly to understanding the role of language, culture and geography in relation to the genetic affiliation and demographic history of Indian populations; a major limitation in them has been the poor representation of Indo-European speaking tribal populations. This is a critically important limitation since the Indo-European speaking tribes provide ample opportunity for examining the influence of linguistic assimilation on the genomic diversity of India.

Keeping the above in view, we present an analysis based on the study of 48 bi-allelic markers in nine Indo-European speaking tribal groups of Southern Gujarat which lies in the western part of India. The main objectives of the study were (a) to study the distribution of Y haplogroups; (b) to study the relative influence of language, geography or ethnicity on the genetic structure of populations using the pattern of Y haplogroup clustering and finally (c) to relate the observed pattern of Y haplogroup clustering with the Y chromosome lineages which arrived in India largely from the Northern corridor at different points of time. To achieve the study objectives the results were first compared with published studies on the Indian populations and then with available data on the Eurasian populations. The Indian populations were selected keeping in view the availability of data, their linguistic and socio-culture status and geographical position. Similarly, the Eurasian populations for which published sources were available were considered for analysis since the historical migrations from these regions are known.

## Materials and Methods

### Collection and processing of blood samples

**Figure 1:**
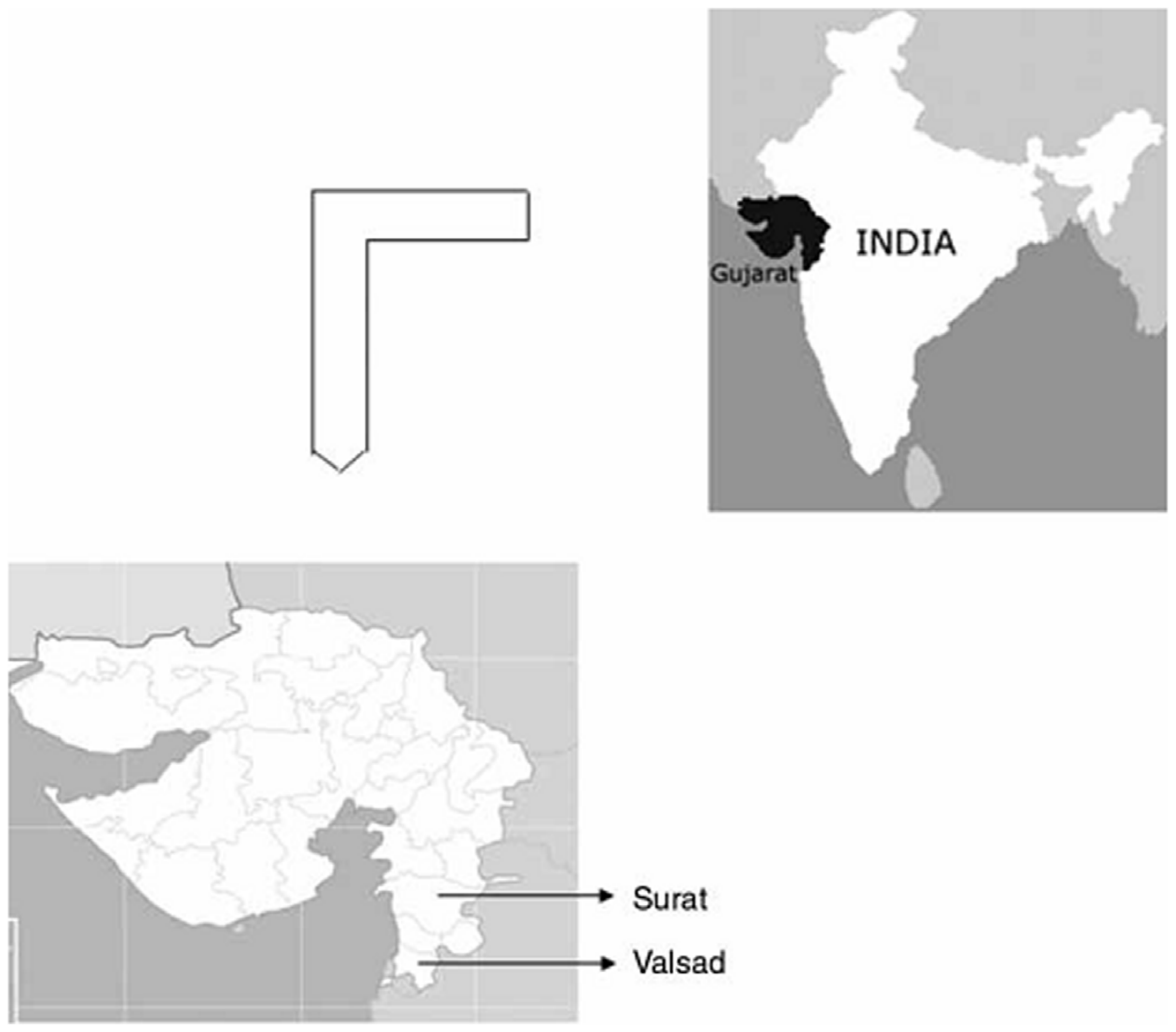
Sampling areas; Map of India highlighting Gujarat (top); Regions of study pointed out in the map of Gujarat (bottom).

This study was approved by the Departmental Ethical Committee of the Department of Anthropology, University of Delhi. Informed written consent was obtained from all the participants. A 5 ml blood sample was drawn by a trained medical practitioner from randomly chosen 284 healthy, unrelated males from nine tribal population groups of Valsad and Surat districts of Gujarat. A description of relevant population characteristicsof study groups along with populations from other studies is presented in Table 1. Figure 1 illustrates the sampling location of study populations from Gujarat. The detailed description of the study populations has been given previously [42]. Blood samples collected in EDTA coated vacutainers were subjected to DNA extraction following salting-out method [43]. A total of 48 bi-allelic markers were analysed to identify the Y chromosome haplogroups. The markers were typed using primer pairs described in Karafet et al. [44]. The PCR cycling conditions with an initial denaturation of 5 minutes at 95°C; followed by 35 cycles of 1 minute at 94°C; 45 seconds at the primer-specific annealing temperature (52–60)°C and extension at 72°C for 2 minutes and 30 seconds; followed by final extension of 7 minutes at 72°C were followed. The amplicons generated were subjected to sequence reactions using BigDye™ (Applied Biosystems, Foster City, USA) Terminator Cycle sequencing kit in ABI Prism 3730 DNA analyser following manufacturer's protocol and further analysed in SeqScape software, version 2.5.

**Table 1:** Geographical, Social and Linguistic description of study groups and populations included in the study for Y haplogroup comparison.

	Population	Linguistic affiliation^a^	Socio-Culture affiliation^b^	Sample size
	**South Asia**			
**India**				
**West**				
	Dhodia	IE	T	63
	Dubla	IE	T	42
	Konkana	IE	T	24
	Vasava	IE	T	24
	Gamit	IE	T	18
	Valvi Chaudhari	IE	T	32
	Nana Chaudhari	IE	T	25
	Mota Chaudhari	IE	T	27
	Pavagadhi Chaudhari	IE	T	29
	Madia Gond	DR	T	14
	Katkari	IE	T	19
	MahadeoKoli	IE	T	11
	Pawara	IE	T	16
	Thakur	IE	T	48
	Desasth Brahmin	IE	C	16
	Maratha	IE	C	16
	Dhangar	IE	C	16
	Chitpavan Brahmin	IE	C	15
	Gujrat Patel	IE	C	9
**South**				
	Chenchu	DR	T	20
	Yerukula	DR	T	18
	Kuruva	DR	T	10
	Irular	DR	T	10
	Naikpod Gonnd	DR	T	18
	Andhra Brahmin	DR	C	15
	Kamma Chaudhary	DR	C	15
	Kappu naidu	DR	C	18
	Komati	DR	C	20
	Raju	DR	C	19
	Reddy	DR	C	12
	Bhovi	DR	C	13
	Gowda	DR	C	4
	Iyengar	DR	C	17
	Lingayat	DR	C	10
	Chakkliar	DR	C	9
	Gounder	DR	C	14
	Kallar	DR	C	9
	Pallar	DR	C	15
	Vanniyar	DR	C	10
**East**				
	Mahali	AA	T	25
	Bhumij	AA	T	15
	Birhor	AA	T	10
	Ho	AA	T	7
	Kharia	AA	T	10
	Munda	AA	T	7
	Santhal	AA	T	7
	Juang	AA	T	10
	Saora	AA	T	13
	Paroja	DR	T	13
	Bhuiyan	IE	T	81
	Bathudi	IE	T	36
	Kora	IE	T	17
	Bihar brahmin	IE	C	18
	kayasth	IE	C	14
	bhumihar	IE	C	20
	Baniya	IE	C	11
	Rajput	IE	C	12
	Yadav	IE	C	8
	Gope	IE	C	16
	Karan	IE	C	18
	Oriya Brahmin	IE	C	24
	Bauri	IE	C	19
	Mahishiya	IE	C	17
	Namasudra	IE	C	13
**Central**				
	Halba	IE	T	21
	jaunsri	IE	T	6
	Bhoksha	IE	C	10
	Kanyakubj Brahmin	IE	C	10
	Khatri	IE	C	7
	Kurmi	IE	C	13
	UP Thakur	IE	C	5
	UP Kurmi	IE	C	6
**North-East**				
	Hamar	TB	T	9
	Kuki	TB	T	7
	Lai	TB	T	10
	Lusei	TB	T	6
	Mara	TB	T	5
**North**				
	Himachal Pradesh RAJPUT	IE	C	15
				
	Afganistan			204
	Pakistan			718
	Iran			150
	**West Asia**			
	Iraq			139
	Jordan			146
	Turkey			523
	Lebanon			104
	Syria			111
	**Central Asia**			
	Kazakhstan			30
	Alltai			98
	Uzbekistan			54
	Kyrgyzthan			13
	Uyghursta			68
	**Europe**			
	Greece			442
	France			23
	Netherlands			27
	Germany			16
	Czech and Slovakia			45
	Alabina			51
	Macedonia			20
	Poland			55
	Hungary			45
	Ukraine			50
	Georgia			63

a Linguistic affiliation: Indo-European (IE), Dravidian (DR), Tibeto-Burman (TB) and Austro-Asiatic (AA);

b Socio-cultural affiliation: Tribe (T), Caste (C).

### Statistical and phylogenetic analysis

The revised Y-Chromosome phylogenetic tree [44] was referred to for the assignment of haplogroups based on informative binary markers. Haplogroup frequencies were estimated by a simple gene count method. In order to determine the genetic structure of study populations, a model propounded by Harpending and Ward [45] was applied. The model examines the relative role of genetic drift and gene flow in causing population differentiation. In the model, the expected frequency of gene diversity for each population is first graphically represented with respect to the distance from the gene frequency centroid, *r_ii_*, which is given by the formula:
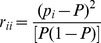
Where, *p_i_* and *P* are the frequency of the haplogroups in a population *i* and in the pooled populations respectively. In the second stage of the model, Harpending and Ward propose an island model of population structure in which there is a linear relationship between gene diversity and the distance from the centroid which is calculated by the formula:
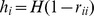
Where, *h_i_* and *H* correspond to the gene diversity value in the population *i* and in all the populations as a whole respectively. As per the model, outlier populations that have undergone systematic migrations will show greater gene diversity than predicted by the regression line, while outlier groups that are isolated will exhibit lower than predicted gene diversity.

In order to determine whether the Y chromosome haplogroup distribution among Indian populations is structured on the basis of ethnicity, geography or linguistic affiliation, an analysis of molecular variance (AMOVA) based on haplogroup frequencies was computed for the various tentative categories using ARLEQUIN software, version 3.1 [46]. Using the same software Slatkin's lineraised pairwise F_ST_ values were calculated on haplogroup frequencies and analysed through non metric multidimensional scaling (MDS) in SPSS version 16.0 using ALSCAL programme. The MDS plot was constructed in order to graphically represent the nature of clustering between the study populations and other world populations.

For carrying out AMOVA on different populations secondary source haplogroup frequency data (Table S1) was compiled from different studies [23]–[25], [47]. For the construction of the MDS plot data (Table S2) was compiled from various studies [48]–[53].

## Results

### Haplogroups distribution

**Figure 2:**
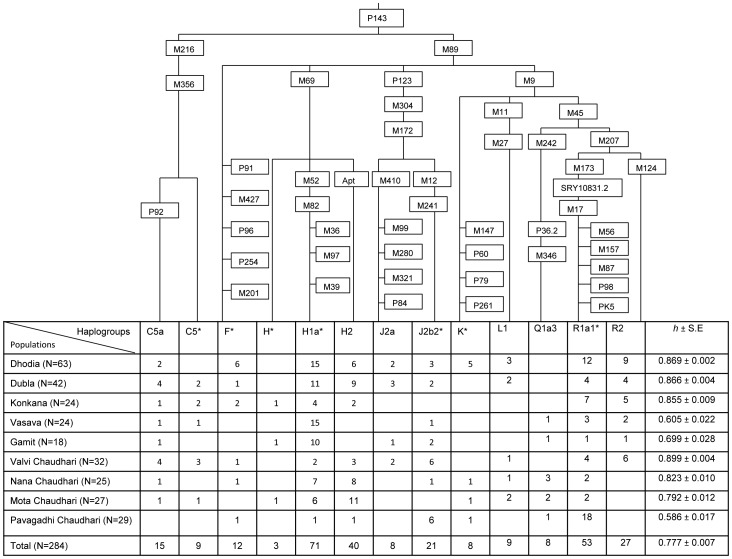
Distribution of Y-binary halpogroups and haplogroup diversity (*h*) among the study populations of Gujarat. The markers used in the study are shown on each branch.

The frequency distribution of Y chromosome haplogroups among the study populations along with the phylogenetic relationship between them is presented in Figure 2. The side branches on the tree represent Y SNP for which the ancestral state was observed, while the direct branch represents Y markers for which the mutant allelic state was observed; leading to haplogroup designation in the particular sample. Analysis of 48 bi-allelic markers of the Y chromosome showed 13 paternal lineages that were distributed throughout haplogroups H, R, J, C, F, L, K and Q. Haplogroup H represented the most frequently occurring haplogroup (40.14%) followed by groups R (28.17%), J (10.21%) and C (8.45%) respectively. Sparse distribution was observed for the lineages F*, L1, Q3 and K* across all the populations.

#### Haplogroup H

M69 mutation, which is a characteristic of haplogroup H was found on 114 of the total 284 Y chromosomes. Haplogroup H was further segregated into three lineages, H1 by the presence of M52-C allele, H2 by the presence of Apt-A allele and H* by absence of the two alleles. These haplogroups were further subdivided into a number of sub-clades. Lineage H1a* of H1 group was observed 71 times and represented the most frequently occurring lineage across all the populations. Its frequency varied from a minimum of 3.45% among Pavagadhi Chaudhari to a maximum of 62.5% in Vasava. Lineage H2 represented the second most frequently occurring lineage among the H haplogroup and third most common haplogroup among all the haplogroups. Its frequency varied from 3.45% among Pavagadhi Chaudhari to 40.74% among Mota Chaudhari. However, H2-Apt was found to be absent among Vasava and Gamit populations. While lineage H* was observed in only three individuals, one each from Konkana, Gamit and Mota Chaudhari.

#### Haplogroup R

R1a1*a sub-clade of R haplogroup was found to be the next most frequently occurring lineage after H1a*. Its frequency was found ranging from 5.56% in Gamit to 62.07% in Pavagadhi Chaudhari. Its sister sub-clade R2-M124 was observed 27 times with a frequency varying from 5.56% among Gamit to 20.83% among the Konkana tribe. Barring Valvi Chaudhari, R2 was absent from all other Chaudhari groups.

#### Haplogroup J

J2 with its two sub-clades J2b2* and J2a constituted a major portion of Haplogroup J in the current study. Except Konkana and Mota Chaudhari, either of the two J2 sub-clades was present in all other groups. J2b2* sub-clade was observed in 21 Y chromosomes. Its frequency was found to be 11% in Gamit, 19% in Valvi Chaudhari and 21% in Pavagadhi Chaudhari. The remaining four populations of Dhodia, Dubla, Vasava and Nana Chaudhari exhibited similar frequency values varying in a narrow range between 4% and 4.48%. Sub-clade J2a was observed only 8 times in the nine groups. It was present in four of the groups with a minimum frequency of 3.17% in Dhodia to a maximum frequency of 7.14% in Dubla.

#### Haplogroup C

Out of the seven sub-clades of C haplogroup, only one sub-clade C5 with its two main derivatives C5a and C5* was observed 21 times in all the populations except Pavagadhi Chaudhari. C5a lineage was observed to be 62 times more frequent then its sister branch C5*. Its frequency varied from 3.17% in Dhodia to 12.5% in Valvi Chaudhari, while that of C5* was found to vary from 3.7% in Mota Chaudhari to 9.38% in Valvi Chaudhari.

#### Haplogroup F*, K*, L1 and Q3

Parahaplogroup F* along with the other parahaplogroup K* and two haplogroups L and Q accounted for 13.03% of the total haplogroups. After initial screening of M89-T allele, 11 samples failed to resolve further and were therefore grouped under F*. Similarly, 8 individuals did not exhibit any mutation except G allele for M9 and were therefore grouped under K*. Other two sub-clades L1-M27 and Q3-M346 were present, but in low frequencies only.

### Y chromosome diversity

Haplogroup diversity values for each of the nine populations along with haplogroup distribution are given in Figure 2. Haplogroup diversity (*h*) which is equivalent to gene diversity for haploid genomes ranged from 0.586 in Pavagadhi Chaudhari to 0.899 in Valvi Chaudhari.

### Population Structure and Gene Flow

**Figure 3:**
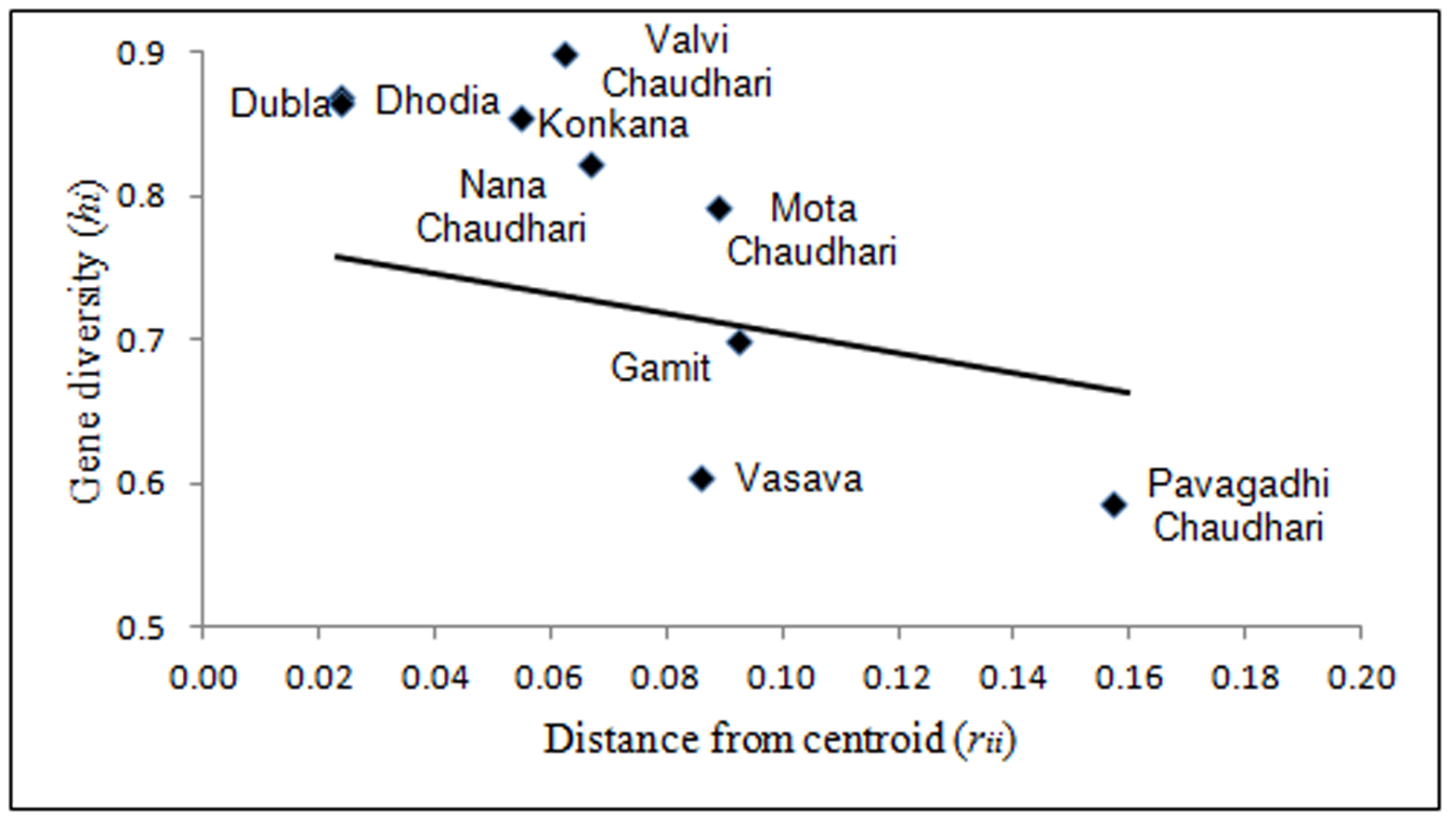
Regression of gene diversity (*h_i_*) on distance from centroid (*r_ii_*). The solid line represents the theoretical regression line.

Figure 3 represents a plot of haplogroup diversity regressed against distance from gene frequency centroid (*r_ii_*). The values of gene diversity (*h_i_*) and genetic distances from centroid (*r_ii_*) used in the nine study population groups along with their standard errors are given in Table 2. Majority of the populations exhibited higher than predicted gene diversity combined with a low to moderate deviation from the theoretical line of regression and the distance from the gene frequency centroid. Three populations Gamit, Vasava and Pavagadhi Chaudhari displayed lower than predicted gene diversity. Pavagadhi Chaudhari showed the farthest distance from the gene frequency centroid. The results indicated that the tribal groups of Gujarat are neither explicitly isolated nor absolutely admixed.

**Table 2:** Gene diversity (*h_i_*) and genetic distances from the centroid (*r_ii_*) among the study populations of Gujarat.

Population	r_ii_ ± S.E	h_i_± S.E
Dhodia	0.024±0.008	0.869±0.002
Dubla	0.024±0.006	0.866±0.004
Konkana	0.055±0.011	0.855±0.009
Vasava	0.086±0.057	0.605±0.022
Gamit	0.092±0.038	0.699±0.028
Valvi Chaudhari	0.062±0.019	0.899±0.004
Nana Chaudhari	0.067±0.029	0.823±0.010
Mota Chaudhari	0.089±0.043	0.792±0.012
Pavagadhi Chaudhari	0.157±0.093	0.586±0.017

### AMOVA

Table 3 presents the results of the AMOVA based on different categories of populations subdivided by language, geography and ethnicity. Analysis of molecular variance based on haplogroup frequencies among the study groups showed that 91.6% of variability is due to within population differences and 8.4% (p<0.001) is due to differences between the populations. In the hierarchical approach taken, at the next level populations were subdivided according to their affiliation into: Indo-European, Dravidian, Tibeto-Burman and Austro-Asiatic language families. A substantially high percentage of difference between populations (13.21%) was observed indicating strong distinctiveness of populations belonging to the different linguistic families. This was followed by the subdivision of populations by the geographical regions they inhabit. The geographical clustering of populations displayed comparatively lower (6.25%) amount of differentiation between the populations. Similarly, in a comparison between Indian castes and tribes a comparatively lower fraction of variance (5.13%) points towards relatively lesser differences between them. Following this, further categories of Indo-European speaking tribal populations of Gujarat were made for comparing with other Indian populations, keeping in mind the effect of language, geography and ethnicity; in that order of importance. The results showed that the lowest group variance among all categories was between Indo-European speaking groups of Gujarat and other Indo-European populations; it was almost 5 times lesser than the variance observed between the studied populations and the Dravidian speaking groups of India. Interestingly, further subdivision of Indo-European populations into tribes and castes revealed a lesser fraction of variability among study populations and Indo-European caste populations (3.45%) as compared to Indo-European speaking tribes (6.57%). Moreover, as compared to Dravidian speaking caste populations, the population in Gujarat showed less differentiation with Dravidian speaking tribes as reflected by low group variance values (5.3%). Subdivision of Indo-European populations simultaneously by caste, tribe and geographical zones (West, Central and East) indicated effective role of geographic distance in determining genetic distance. All the values compared were found to be highly significant.

**Table 3:** AMOVA based on Y Chromosome haplogroup frequencies.

Categories	Among groups variance (%)	Among populations within groups variance (%)	Within populations variance (%)
IE speaking tribes of Gujarat	8.40		91.60
4 language groups^a^	13.21	18.52	68.27
Geography^b^	6.25	23.22	70.53
Castes and tribes of India	5.13	24.70	70.17
IE speaking tribes of Gujarat with IE populations of India	1.81	19.57	78.62
IE speaking tribes of Gujarat with DR populations of India	9.61	11.55	78.84
IE speaking tribes of Gujarat with AA Populations of India	28.16	7.94	63.90
IE speaking tribes of Gujarat with IE castes of India	3.45	12.97	83.58
IE speaking tribes of Gujarat with IE tribes of India	6.57	15.17	78.26
IE speaking tribes of Gujarat with DR tribes of India	5.30	11.47	83.23
IE speaking tribes of Gujarat with DR caste of India	8.54	12.51	78.95
IE speaking tribes of Gujarat with IE tribes from three geographical regions C,W & E of India	9.24	15.12	75.63
IE speaking tribes of Gujarat with IE castes from three geographical regions C,W & E of India	7.59	9.78	82.63

a Language groups = Indo-European (IE), Dravidian (DR), Tibeto-Burman (TB) and Austro-Asiatic (AA),

b Geography = Central (C), West (W), East (E), South(S) and North East (NE). All the values are significant, p<0.05.

### Genetic proximities

**Figure 4:**
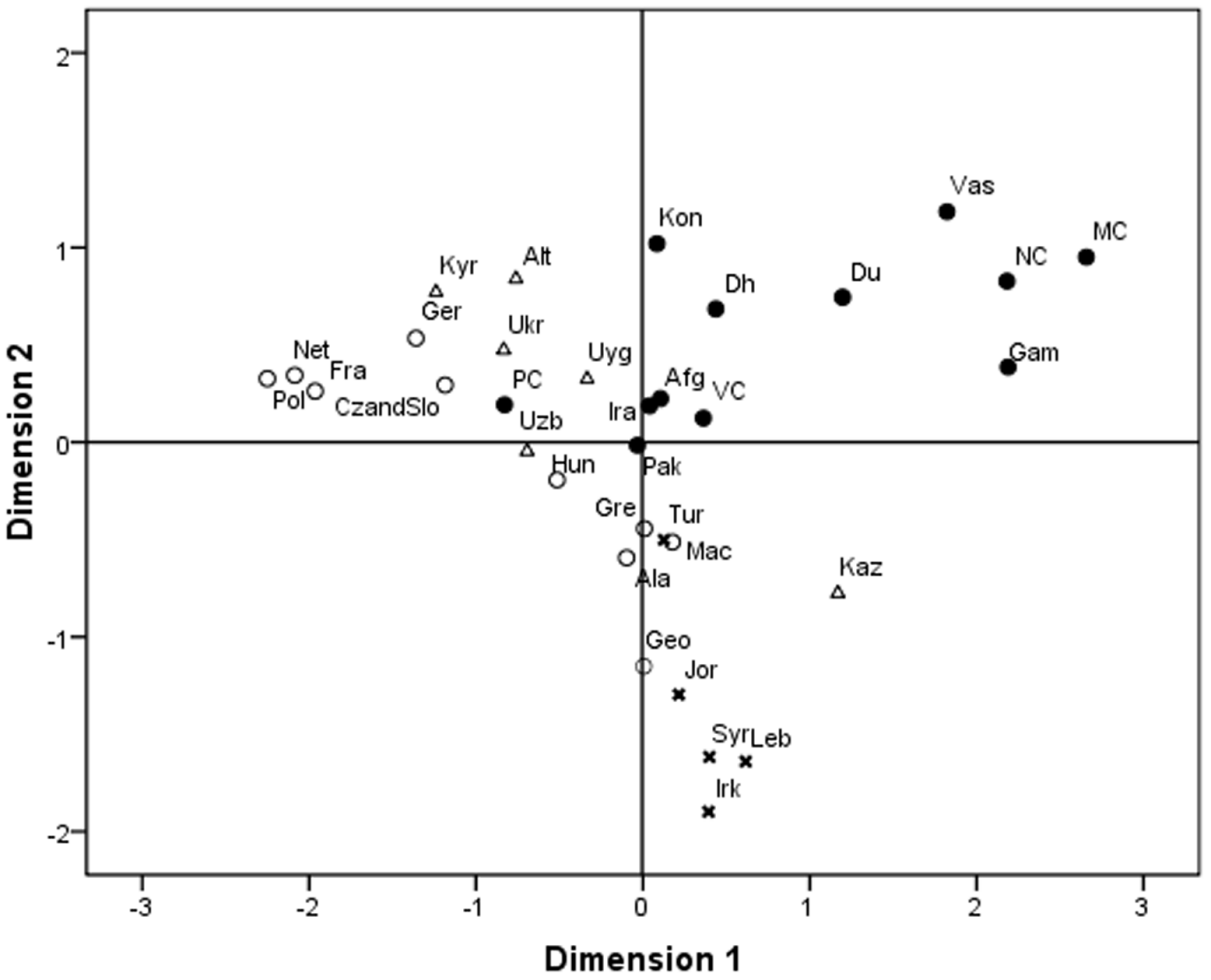
MDS Plot showing genetic relationships particularly between the South Asian populations with the world populations. The South Asian populations including the study populations of India are shown in as solid circles (•), the European populations as open circles (o), Central Asian populations as triangle (Δ) and West Asian population as cross (x). The abbreviation used are Afganistan (Afg), Pakistan (Pak), Iran (Ira), Iraq (Irk), Jordan (Jor), Turkey (Tur), Lebanon (Leb), Syria (Syr), Kazakhstan (Kaz), Altai (Alt), Uzbekistan (Uzb), Kyrgyztan (Kyr), Uyghurstan (Uyg), Greece (Gre), France (Fra), Netherlands (Net), Germany (Ger), Czech and Slovakia (CzandSlo), Alabina (Ala), Macedonia (Mac), Poland (Pol), Hungary (Hun), Ukraine (Ukr), Georgia (Geo), Dhodia (Dh), Dubla (Du), Konkana (Kon), Vasava (Vas), Gamit (Gam), Valvi Chaudhari (VC), Nana Chaudhari (NC), Mota Chaudhari (MC), Pavagadhi Chaudhari (PC).

We compared the study populations with 24 additional world populations already published in separate studies (Figure 4). A stress value of 0.18 for the MDS plot indicated a good fit between the two dimensional graph and the original distance matrix. The comparison revealed four major clusters of South Asian, Central Asian, West Asian and European populations. All the populations under study with the exception of Pavagadhi Chaudhari were found to be clustered together. The occurrence of South Asian populations (the current study groups, Afghanistan, Pakistan and Iran) with Central Asian populations (Kazakhstan, Altai Region, Uzbekistan, Kyrgyzstan, Uyghurstan) on Axis I, conversely with West Asian populations (Iraq, Jordan, Turkey, Lebanon, Syria) with respect to Axis II probably indicate similarities of haplogroups between them.

## Discussion

The North-Western corridor of India has witnessed many waves of migrants from different parts of the world, with a majority being male migrants [31]. Gujarat is located on the western most point of the Indian sub-continent and has acted as a significant corridor to draw outsiders -conquerors, refugees and travellers who have contributed significantly to the present day gene pool of Indian populations [34]. Valsad and Surat districts are part of the tribal belt of Gujarat and are composed of tribal populations which are linguistically classified as Indo-European. To date several studies have provided significant insights into the paternal genetic history of India [23]–[24], [34], [36]; however none till now had taken into consideration the Indo-European speaking tribal populations inhabiting Gujarat in spite of their interesting geographical location and cultural attributes. It is also worth mentioning that the Indo-European speaking tribes of India not only exhibit the complexity of historical interaction between the indigenous Indian and migratory groups but also reflect lack of one-to-one correlation between language, mode of subsistence and social system [23]. Thus, the Indo-European speaking tribes represent an appropriate model to study the possible genetic foot prints of the multiple waves of migrations. In addition to tracing the origin and impact of ancient migrations, evaluation of the impact of geography and social structure in shaping the genetic structure of the present day Indo-European speaking tribal populations of Gujarat was considered equally important. Thus, in the subsequent text we examine the paternal genetic variation of the nine Indo-European speaking tribes from Gujarat using high resolution Y chromosomal unique event polymorphisms (UEPs).

### Genetic structure of study populations

Our analysis based on Y chromosomal UEPs apportioned the study samples into 13 haplogroups representing 10 major Y lineages (C5, H1a*, H2, R1a1*, R2, J2, L1, F*, K* and Q3) in India. Haplogroup diversity (0.772) was found to be comparable with Dravidian speaking populations (0.723), but higher than Indo-European speaking populations (0.684) in the country [54]. Given the number of views in support of Austro-Asiatic and Dravidian speaking tribes belonging to the primary pre-historic population of India [7], [11]; the high gene diversity values observed in the study populations, which are in turn comparable to Dravidian speaking populations, are suggestive of greater antiquity, large effective size or role of gene flow in these groups. The analysis of molecular variance revealed that the extent of genetic differentiation was high among study populations which could be attributed to either the lower effective population size of these groups or the Y chromosome making them vulnerable to the effect of genetic drift which further accelerates the process of differentiation between the populations. But, accentuated differentiation due to genetic drift is expected to be accompanied with lower diversity. However, in the present study, elevated levels of gene diversity, rule out a major role of genetic drift in shaping the observed pattern of genetic differentiation. Nevertheless, the explanation for the observation of high gene diversity coupled with high-level of genetic differentiation could lie in an overwhelming genetic admixture from different sources or in an early inflow of genes from a common source followed by rapid population expansion and subsequent fission into isolated, endogamous populations. Results from Harpending and Ward analysis do not appear to favour the former explanation. Thus, the most plausible explanation for the current diversity scenario in the Indian population seems to be the suggestion made by Majumder et al. [17], in which an early demographic expansion of modern humans within India during Palaeolithic period and later fission of the populations is conjectured.

### In-situ versus Ex-situ Origin of Y lineages

The indigenous versus exogenous origin of Indian paternal lineages has been widely contested. Among the study populations, six sub-haplogroups namely, C5, H1a*, H2, J2, R1a1* and R2 constituted the major paternal lineages that together accounted for 85.92% of the Y chromosomes. While the indigenous origin of sub-clades C5, H1a*, H2 and R2 are accepted, the status of sub-clades J2 and R1a1* are contested as they are believed to have been introduced in India with the demic diffusion of Proto-Dravidian Neolithic agriculturists from West Asia and the influx of Indo-European pastorals from Central Asia. It is worth mentioning here that the high frequency and associated diversity of a haplogroup is correlated with the possible place of origin of a particular haplogroup [55]. In the present investigation haplogroup H, especially H1a, represented the most frequently observed Y chromosomal lineage followed by H2 sub-haplogroup. Its higher frequency among the Indian tribes particularly among the Dravidian speaking tribes of South India and its limited presence elsewhere on the Indian subcontinent had led some scholars to denote it as a tribe-specific haplogroup [36]. However, several subsequent studies have confirmed the presence and equal prevalence of haplogroup H and its associated H1a and H2 branches across linguistically and ethnically diverse populations and in different regions of India, except the North-Eastern region [23]–[24], [54]; thus ruling it out as a tribal-specific marker and supporting the uniform distribution of haplogroup H among Indian populations. An Indian homeland for haplogroup H can also not be refuted keeping in view its higher microsatellite diversity among Indian populations [24]. Haplogroup H has also been reported from Central Asian, West Asian and Gypsy populations in Europe. However, its low frequency and prevalent diversity pattern in Central Asia and West Asia could be due to recent back migration [32], [52]. On the other hand, the established Indian ancestry of gypsy populations is surely the reason for elevated levels of H haplogroup among them [56]. All the observations, therefore clearly point towards in-situ origin of haplogroup H among Indian populations.

Haplogroup C is widely distributed in Eastern and Central Asia, Oceania and Australia [44]. As expected, all the Y chromosomes under C haplogroup belonged to the C5 sub-clade. It was observed in a frequency of 8.45% which is the highest ever reported frequency for C5 in India and whose spread is circumscribed along the coastal belt of India [24], [54]. High STR diversity in India and South-East Asia in the backdrop of haplogroup C has also been observed [34], [57]. Interestingly none of the C haplogroup derivatives frequent in South-East Asia have been reported from India. Therefore, the possibility of introduction of C5 haplogroup from South-East Asia as a result of back migration to India appears doubtful and its indigenous origin appears to be more probable [13].

Haplogroup J is predominantly found among the populations of West Asia, North Africa, Europe, Central Asia, Pakistan, and India [44] and widely linked with the spread of agriculture from the Fertile Crescent that extends from Israel to Western Iraq. In the Indian subcontinent two sub-clades of J - J2a and J2b have been reported. Consistent with the previous studies, a higher proportion of J2 in West India as compared to North and South regions of India has been recorded in the present study [24], [54]. Two models pertaining to the homeland for Indian J2 sub-clades have been contested, West Asia and Central Asia. Cordaux et al. [35] had proposed the Central Asian homeland for Indian J2 sub-clade mainly because of higher frequency of J2 in Central Asia. It is worth mentioning here that J1 sub-clade which appears in appreciable frequency in Central Asia is largely absent from the Indian as well as most of the West Asian populations [44]. Haplogroup R is represented by two sub-clades R1a1* and R2 among the study populations. After haplogroup H1a, haplogroup R1a1* represented the most frequently occurring haplogroup. Haplogroup R is widely distributed in Central Asia, Eastern Europe, West Asia and the Indian subcontinent [58]–[59]. The higher frequency of haplogroup R1a, up to 63% in Central Asia and its relatively lower occurrence in other regions has been linked with Central Asian origin of R1a clade [36]. However, later studies [24], [54] showing higher prevalence of R1a sub-clade along with high microsatellite diversity among the tribal populations of India lend support to probable South Asian origin of R1a sub-clade as suggested by Kivisild et al. [34]. This is further substantiated by almost the complete absence of other derivatives of haplogroup R1 among the Indian populations, which is expected in case of the inflow from Central Asia [23]–[24], [41].

In the present investigation sub-clade R2 occurred with a frequency of 9.51%, which is similar to its frequency reported from other Indian populations [24], [34], [36], [54]. The frequency of R2 decreases as one goes further west from India and its frequency is almost negligible in Europe. Moreover, its frequent occurrence among Dravidian speaking groups as compared to Indo-European or Austro-Asiatic speaking groups of India can be attributed to its indigenous Indian origin.

The MDS plot (Figure 4) also reflects the closeness of South Asian populations with West Asian and Central Asian populations possibly due to overlapping of haplogroups. In comparison to the world populations the overall mean haplogroup diversity among the study populations was relatively higher than in the European or East Asian populations [32], [48] whereas it was found to be lower than that of Central Asian and West Asian populations [49], [51]. As mentioned earlier the high frequency and diversity of a haplogroup is indicator of the possible place of origin of a particular haplogroup [55]. Consequently, the observed haplogroups in the present investigation could be apportioned into three Y lineages based on their possible origin. These lineages include Central Asian, West Asian and indigenous Indian Y lineages.

### Determinants of population Clustering

Lack of any major exogenous contribution of Y lineages and existence of high haplogroup diversity indicate the possible male assimilation from other neighbouring populations of India among the study groups. Analysis of molecular variance (Table 3) performed on the basis of haplogroup frequencies revealed some interesting patterns. The quantification of variance into different categories of population showed the highest variance between the four linguistic groups of India followed by the variance between geographical regions and finally the caste versus tribe categories. The least percentage of variance was observed between the study populations and other Indo-European speaking caste populations. However, the geographical partitioning of the Indo-European speaking tribal and caste populations into three major zones: West, Central and East showed the important effect of geography in shaping the Y diversity pattern since on this basis the zonal percentage of variance was observed to elevate between the study populations and other Indo-European groups of India. Thus, pattern of Y chromosome clustering of Indian populations reflects the major role of geography over language and ethnicity as far as Y chromosomal lineages are concerned. After Indo-European speaking caste populations, the lowest variance of Gujarat populations was observed with Dravidian speaking tribal populations. These similarities suggest either shared paternal ancestries of linguistically dissimilar groups or the influence over the indigenous tribal groups of India of the Indo-European speakers, who arrived later.

The present investigation indicates that tribes of Gujarat show both high genetic diversity and genetic differentiation. It is therefore conceivable that these groups, with a fairly large population size, have passed through a long evolutionary history experiencing an early demographic expansion and later fission of the populations. Further affinities of study groups with Indo-European speaking non tribes followed by Dravidian speaking tribal groups suggest the possibility that these native tribal population groups of Western India might have adopted the Indo-European language during the process of cultural assimilation and absorption while still retaining their genetic links with the Dravidian speaking tribal populations. Thus, it is recommended that the present day linguistic affiliation of any Indian population should be considered with caution while reconstructing the demographic history of the country. In conclusion, a study based on the recently discovered bi-allelic loci and microsatellite loci in the populations can shed light on the possible explanation of the overlapping of haplogroup distribution between tribes of Gujarat with other Asian populations and further deepen the understanding of the population history of India.

## Supporting Information

Table S1 **Y chromosome haplogroup frequencies data among study populations and other populations of India considered for AMOVA.**
(XLS)

Table S2 **Y chromosome haplogroup frequencies data among study populations and other populations of World considered for MDS analyses.**
(XLS)
